# Differential Regulation of 6- and 7-Transmembrane Helix Variants of μ-Opioid Receptor in Response to Morphine Stimulation

**DOI:** 10.1371/journal.pone.0142826

**Published:** 2015-11-10

**Authors:** Marino Convertino, Alexander Samoshkin, Chi T. Viet, Josee Gauthier, Steven P. Li Fraine, Reza Sharif-Naeini, Brian L. Schmidt, William Maixner, Luda Diatchenko, Nikolay V. Dokholyan

**Affiliations:** 1 Biochemistry and Biophysics Department, University of North Carolina, 120 Mason Farm Road, Chapel Hill, NC, United States of America, 27599; 2 Alan Edwards Centre for Research on Pain, McGill University, 740 Dr. Penfield Avenue, Montreal, Quebec, Canada, H3A 0G1; 3 Bluestone Center for Clinic Research, New York University, New York, NY, United States of America, 10010; 4 Center for Pain Research and Innovation, University of North Carolina, 385 S. Columbia Street, Chapel Hill, NC, United States of America, 27599; 5 Department of Physiology and Cell Information Systems, McGill University, 3649 Promenade Sir William Osler, Montreal, Quebec, Canada, H3G 0B1; Temple University, UNITED STATES

## Abstract

The pharmacological effect of opioids originates, at the cellular level, by their interaction with the μ-opioid receptor (mOR) resulting in the regulation of voltage-gated Ca^2+^ channels and inwardly rectifying K^+^ channels that ultimately modulate the synaptic transmission. Recently, an alternative six trans-membrane helix isoform of mOR, (6TM-mOR) has been identified, but its function and signaling are still largely unknown. Here, we present the structural and functional mechanisms of 6TM-mOR signaling activity upon binding to morphine. Our data suggest that despite the similarity of binding modes of the alternative 6TM-mOR and the dominant seven trans-membrane helix variant (7TM-mOR), the interaction with morphine generates different dynamic responses in the two receptors, thus, promoting the activation of different mOR-specific signaling pathways. We characterize a series of 6TM-mOR-specific cellular responses, and observed that they are significantly different from those for 7TM-mOR. Morphine stimulation of 6TM-mOR does not promote a cellular cAMP response, while it increases the intracellular Ca^2+^ concentration and reduces the cellular K^+^ conductance. Our findings indicate that 6TM-mOR has a unique contribution to the cellular opioid responses. Therefore, it should be considered as a relevant target for the development of novel pharmacological tools and medical protocols involving the use of opioids.

## Introduction

Opioid analgesics are the most prescribed and effective drugs for the treatment of moderate and severe pain [1]. The majority of opioid analgesics are agonists of the μ-opioid receptor (mOR), a G protein-coupled receptor (GPCR) that modulates the basal antinociceptive response [2–4]. The analgesic effect of opioids is associated with the mOR-mediated activation of Gα_i/o_ protein, which promotes the reduction of intracellular concentration of cyclic adenosine monophosphate (cAMP) and intracellular Ca^2+^, while it stimulates an increase in cellular K^+^ conductance, and ultimately leads to the inhibition of the synaptic transmission [5,6]. This pharmacological response is generated by the interaction of opioid analgesics with the mOR binding site that fosters a conformational rearrangement of the receptor’s intracellular loops, and consequently mediates the activation of the Gα_i/o_ protein [7–10]. Furthermore, ligand stimulation of mOR activates non-canonical signaling pathways that include receptor phosphorylation by GPCR kinases (GRKs), and interactions with β-arrestins, which, consequently, triggers receptor internalization process, and determines mOR down-regulation and resensitization [11–14].

Here, we report the structural and functional mechanism of a recently discovered spliced variant of mOR, namely mOR-1K, consisting of six trans-membrane helices (6TM-mOR) [15–18], that may contributes to the development of a cellular excitatory signaling upon activation with morphine, the most clinically relevant mOR agonist. We demonstrate that, differently from what has been observed in the wild type seven trans-membrane helix mOR (7TM-mOR), the binding of morphine to 6TM-mOR does not induce the same dynamic response of the mOR third intracellular (i3) loop, which is directly involved in the interaction with Gα_i/o_ protein [7–10]. Consequently, the stimulation of 6TM-mOR with morphine does not induce any intracellular cAMP response. Furthermore, we show that, upon binding to morphine, 6TM-mOR-expressing cells are characterized by a decreased K^+^ conductance, and increased intracellular Ca^2+^ concentration, which are associated with excitatory cellular response [6].

## Results and Discussion

### Morphine binding differentiates 6TM- and 7TM-mOR dynamic properties

**Fig 1:**
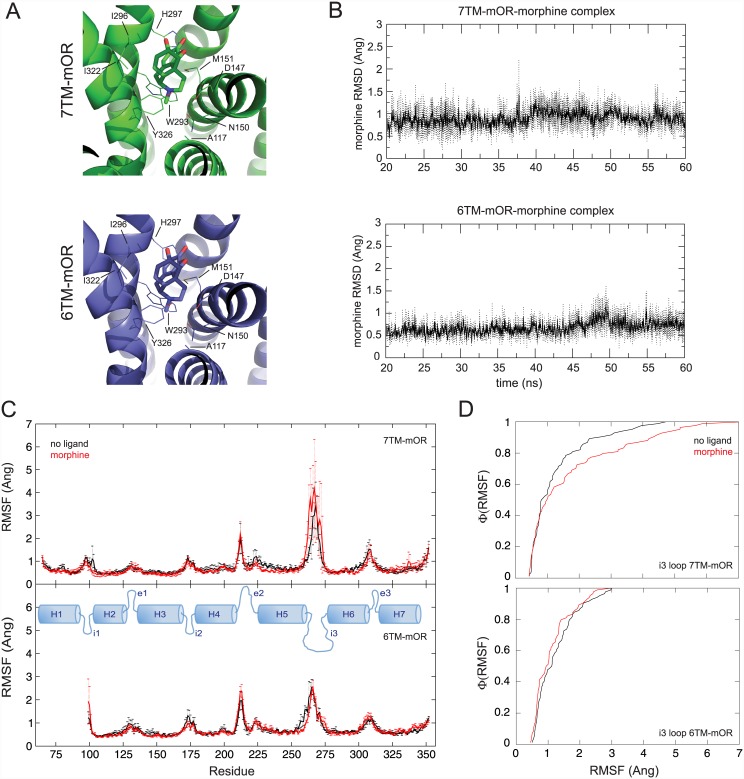
Structure and dynamics of 7TM- and 6TM-mOR in complex with morphine. **A)** The binding modes of morphine in 7TM- (green) and 6TM-mOR (blue) as obtained with MedusaDock are reported on the left. The energy of binding, estimated using MedusaScore, is equal to -40.3 kcal/mol and -39.5 kcal/mol for 7TM-mOR and 6TM-mOR, respectively. The RMSD of superimposed conformations is ~ 0.3 Å. **B)** The identified binding mode is very stable as shown by the RMSD time series computed on the heavy atoms of morphine obtained from three independent MD simulations of the two morphine-bound isoforms. **C)** Fluctuations of Cα atoms between the apo- (black) and morphine-bound (red) receptors. The binding of morphine increases the fluctuations of 7TM-mOR i3 loop, which is known to interact with the G protein (top). 6TM-mOR seems to be more stable than the 7TM-isoform, and the binding of morphine does not affect the dynamic fluctuations of the i3 loop as the in 7TM-mOR isoform (low). Data are presented as mean ± standard deviation from three independent MD simulations. The area under the curve values calculated over the average root mean square fluctuations (RMSF) for residues 256–283 (*i*.*e*., i3) are 35.02 and 47.77 for morphine-free and morphine-bound 7TM-mOR, respectively, and 33.85 and 30.57 for free and morphine-bound 6TM-mOR, respectively. Topology map of mOR indicating helices (H), intracellular (i), and extracellular (e) loop is reported in blue. **D)** RMSF cumulative distributions of i3 loop amino acids are different in apo- and morphine-bound conformation of 7TM- (top) and 6TM-mOR (botton) with a p-value of 0.19 and 0.36, respectively as determined with the Kolmogorov-Smirnov test [31]. Apo- and morphine-bound conformation data are reported as black and red lines, respectively. Analyses are performed on the last 40 ns of simulations of three independent simulations.

In order to elucidate the structural and functional difference between 6TM- and 7TM-mOR at the molecular level, we explore the binding properties of morphine in both mOR variants. We model the structures of the two proteins using discrete molecular dynamics simulations [19,20] starting from the crystallographic coordinates of the mOR chimera [21]. Then, we perform docking calculations with MedusaDock [22,23], which properly accounts for the induced-fit phenomenon and eliminates the possible bias originated from the starting antagonist-bound conformation of the amino acids in the GPCR binding pockets. In both 7TM- and 6TM-mOR, the identified bound conformation of morphine participates in van der Waals interactions with A117, M151, W293, I296, I322, G325, and is involved in polar interactions with D147, N150, Y326, and H297 (Fig 1, panel A), consistently with previously published data [24]. The binding energy as estimated by MedusaScore [25] of morphine in 7TM- and 6TM-mOR is -40.3 kcal/mol and -39.5 kcal/mol, respectively. Binding interactions are very stable in both mOR isoforms as computationally determined by the atomic distance between morphine and amino acids in the orthosteric binding site of mOR isoforms (S1 and S2 Figs). In 7TM- and 6TM-mOR, the bound conformation of morphine persists respectively within an average of ~ 0.9 Å and ~ 0.7 Å from the initial docking conformations (Panel A S3 Fig), and the root mean square distance (RMSD) between morphine bound conformations in the two receptors after 60-ns simulations is equal to ~ 1.3 Å (Panel B S3 Fig). These results suggest that the binding mode of morphine in 7TM- and 6TM-mOR are energetically and geometrically very similar as also revealed by RMSD time series of morphine’s heavy atoms computed over three independent 60-ns long molecular dynamics simulations (Fig 1, panel B). Our *in silico* data indicate that morphine may interact with the opioid orthosteric binding site in both 6TM- and in 7TM-mOR. This scenario is consistent with the lack of competition observed in 6TM-mOR between morphine and iodobenzoylnaltrexamide (IBNTxA) or iodobenzoylnaloxamide [26,27], which may bind to alternative 6TM-mOR sites that become accessible upon heterodimerization of 6TM-mOR with a second GPCR partner [26]. Dinstict experiments performed by different research groups [26,28] indicate a *K*_*i*_ values for morphine towards 7TM-mMOR of 1.8 nM (measured in competition assay with [D-Ala^2^,N-Me-Phe^4^,Gly^5^-ol]enkephalin (DAMGO)). On the other hand, *K*_*i*_ of morphine towards 6TM-mMOR is greater than 1μM (measured in competition assay with IBNTxA in 6TM-mOR). The high diversity of the *K*_*i*_ values may be related to the specific competition assays, as well as the peculiar properties of IBNTxA, which has shown binding to 6TM-mOR only when the protein is co-expressed with nociceptin. [26]. Indeed, it has been recently demonstrated that 6TM-mOR can heterodimerize with a second GPCR, like nociceptin [26] or β2-adrenergic receptor (β2AR) [18]. Thus, we cannot exclude that the affinity, potency, and isoform-specificity of morphinans towards 6TM-mOR can vary according to the specific oligomeric state of the receptors and the distinct GPCR partner involved in 6TM-mOR oligomerization.

Despite the similarity of binding modes observed in our simulations, the interaction with morphine differentiates the dynamic properties of 7TM- and 6TM-mOR’s i3 loops. Indeed, amino acids in the i3 loop show the tendency of having higher dynamic fluctations in 7TM-, but not in 6TM-mOR complex (Fig 1 panels C and D). Residues in the i3 loop of GPCRs directly interact with G proteins [8], which are responsible for the initiation of the intracellular signaling processes [9]. Although, it has been shown that GPCR conformational transitions, involving the i3 loop and resulting in the activation/inactivation of the receptor, occur in the microsecond scale [29,30], we speculate that the tendency of increased dynamic fluctuations of morphine-bound 7TM-mOR’s i3 loop amino acids, observed in our simulations (tens of nanoseconds time scale), may lower the free energy barrier of activation of the receptor as a consequence of increased entropic contributions. Our data also suggest that the binding of morphine to 6TM-mOR may not promote the same dynamic fluctuations of amino acids in the 6TM-mOR’s i3 loop, as observed in the major mOR isoform. Under this scenario, the interaction between the i3 loop of 6TM-mOR and the G protein might be potentially compromised as well as the classic G protein-dependent intracellular signaling cascade. Overall, our computational findings suggest that both mOR isoforms can interact with morphine. Nevertheless, upon binding of morphine, 6TM-mOR does not produce the same dynamic response as 7TM-mOR, and, thus, it may not activate a similar cellular response.

### Morphine binding to 6TM-mOR does not promote cAMP response

In order to elucidate how the different dynamics of 6TM- and 7TM-mOR upon binding of morphine affect the opioid signaling pathways, we investigated the specific cellular response to the exposure of this drug. The absence of a morphine-mediated increase in the dynamics of the i3 loop of 6TM-mOR suggests that the binding of morphine to 6TM-mOR may perturb the formation of the 6TM-mOR-Gα_i/o_ complex, and the subsequent inhibition of adenylate cyclase (AC). The inhibition of AC is characteristic for the wild type 7TM-mOR, [5,32] and has been implicated in the suppression of neuronal activity [6]. Previous studies showed that the stimulation of 6TM-mOR with 1 μM of morphine does not promote the decrease of intracellular forskolin-induced cAMP levels in COS1 mamalian cells [16] as observed for the wild type 7TM-mOR. However, since morphine dose-response testing has not been done, there is possibility that morphine produces inhibition of AC in the 6TM-mOR dependent manner at the concentrations higher than 1 μM. To quantitatively test this hypothesis, we perform a cAMP-sensitive luciferase reporter assay, which measures the morphine dose-dependent levels of intracellular cAMP.

**Fig 2:**
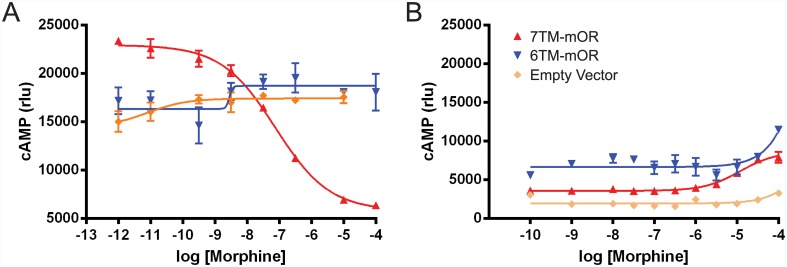
7TM-mOR- and 6TM-mOR-dependent cAMP regulation. HEK293 cells transiently transfected with 7TM-, 6TM-mOR and empty vector (control) expressing constructs are subjected to cAMP-sensitive luciferase reporter assay. Increasing concentrations of morphine are applied either to isoproterenol (100 nM, for 5 min) pre-treated cells (A) or to untreated cells (B) to detect inhibition of cAMP production within 30 minutes. Data are analyzed with Prism software (GraphPad, Software, San Diego, CA). Mean ± S.E.M., n = 3 in triplicate.

Luciferase reporter assay confirmed that increasing concentrations of morphine, ranging from 10^−12^ to 10^−4^ M, do not yield any change in cAMP levels in human embryonic kidney (HEK293) cells, transfected with 6TM-mOR, neither in the conditions of stimulated cAMP levels with 100 nM of isoproterenol (to detect ligand-dependent inhibition of cAMP levels, Fig 2, panel A), nor in unstimulated cells (to detect ligand-dependent stimulation of cAMP levels, Fig 2, panel B). Although at the highest concentrations of morphine we observe a slight increase in cAMP production in 6TM-mOR transfected cells (Fig 2), we also observe a comparable increase in 7TM-mOR transfected cells or empty vector transfected cells. The latter observation makes us conclude that this morphine effect is not opioid receptor specific and, importantly, not 6TM-mOR dependent. On the contrary, 7TM-mOR transfected cells show robust inhibition of isoprotenerol-stimulated cAMP levels (Fig 2, panel A) at IC_50_ of 80 nM. This is in accordance with previous literature reporting IC_50_ values in a range of 10^−8^–10^−7^ M [33–35]. Together, these data suggest that, in contrast to 7TM-mOR that produces robust drug-dependent cAMP inhibition, the stimulation of 6TM-mOR does not result in a morphine-mediated intracellular response.

### Stimulation of 6TM-mOR increases the intracellular Ca^2+^ concentration

**Fig 3:**
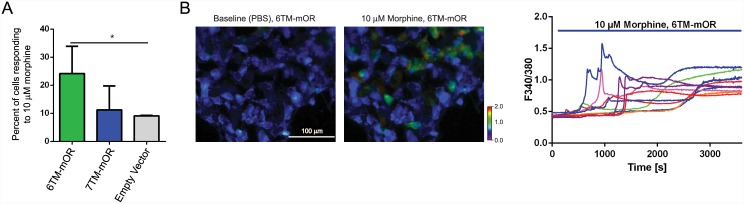
6TM-mOR transfected Be2C cells show positive Ca^2+^ response to the stimulation with morphine. **(A)** The percentage of Ca^2+^-responding cells upon 10 μM morphine treatment. Be2C cells transfected with 6TM-mOR, 7TM-mOR, or empty vector plasmids. *p<0.03, one-way ANOVA and Fisher’s LSD. Perfusion conditions: 1 min PBS; 59 min 10 μM morphine. **(B)** Time lapse images of Be2C cells transfected with 6TM-MOR at baseline (left; perfused with PBS) and after morphine treatment (right; perfused with 10 μM morphine). Ca^2+^ responses are imaged using Fura-2AM ratio-metric dye. Color scale shows the F340/380 fluorescence intensity ratio increase. Scale bar: 100 μm. **(C)** Ca^2+^ responses of individual 6TM-mOR-transfected cells, presented as increases in F340/380 fluorescence intensity; 10 μM morphine was perfused between 60–3600 s (blue bar).

Morphine-mediated cellular response results in the inhibition of pre- and postsynaptic voltage-gated Ca^2+^ channels (VGCC) [36]. Therefore, we investigate morphine-dependent dynamics of the intracellular concentration of Ca^2+^ to further characterize the 6TM-mOR-dependent cellular response. We have previously shown that the stimulation of 6TM-mOR results in a morphine-dependent increase in intracellular Ca^2+^ response in dose dependent manner, with administration of 10μM of morphine resulting in greatest increase [16]. Here, we monitor the morphine-dependent intracellular Ca^2+^ upon stimulation with morphine of both 7TM-mOR and 6TM-mOR isoforms dynamics in human neuroblastoma Be2C cell line using real time Ca^2+^ imaging (Fig 3). In morphine dose-response studies we first establish that morphine initiates detectable Ca^2+^ release at 1μM, gradually reaching a plateau. We then use 10μM of morphine for all Ca^2+^ assays and electrophysiological experiments to stay at the plateau of cellular response. A high concentration of morphine is needed for the activation of 6TM-mOR, in line with a high *K*_*i*_ for morphine binding in 7TM-mOR knock-out mice [26]. Using this technique, we confirm a stable baseline before application of morphine (proving that we are not recording spontaneous cellular activity), and get a better understanding of the kinetics of Ca^2+^ influx. During a one-hour exposure to morphine, both the percentage of responders (Fig 3, panels A and B) and the amplitude of Ca^2+^ response (Fig 3, panel C) are increased in cells expressing 6TM-mOR, while cells expressing 7TM-mOR show a Ca^2+^ response similar to the control (*i*.*e*., empty vector). Given that the percentage of responding 7TM-mOR-transfected cells is similar to control, we conclude that induction of Ca^2+^ current can derive from endogenous 6TM-mOR expression, which is relatively high in Be2C cells [16]. Furthermore, we observe that the morphine-dependent Ca^2+^ response is not immediate, but it gradually increases with the first spikes appearing at 500s (Fig 3, panel C), likely indicating a VGCC activation, secondary to 6TM-mOR stimulation. Given the lack of cAMP response observed upon 6TM-mOR stimulation, the later Ca^2+^ response may derive from the signaling activity of 6TM-mOR in its heterodimeric form with other GPCRs, such as nocicpetin (Pasternak) or β2AR (Samoshkin), however this hypothesis would require futher investagation. Overall, these results characterize a 6TM-mOR-dependent Ca^2+^ response, and reveal an increased morphine-dependent Ca^2+^ response under unstimulated Ca^2+^ level condition, while stimulation of 7TM-mOR under this condition produces no response.

### Stimulation of 6TM-mOR reduces cellular K^+^ conductance

**Fig 4:**
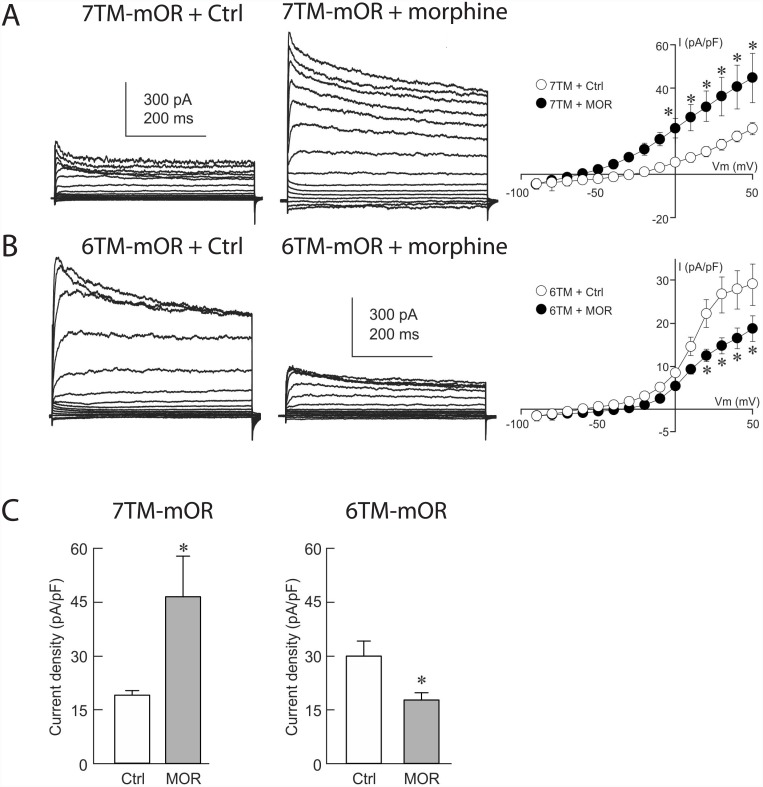
Electrophysiological response of Be2C cells in response to stimulation with morphine. **A)** Morphine stimulation of Be2C cells transfected with the 7TM-mOR causes a rise in an outward current with a reversal potential near -80 mV, suggesting that K^+^ conductance is enhanced. **B)** Morphine stimulation of Be2C cells transfected with the 6TM-mOR leads to a decrease in an outward current with a reversal potential near -80 mV, suggesting that K^+^ conductance is reduced. **C)** Averaged current density data obtained at +50 mV indicates an increase in current density in response to morphine in 7TM-mOR-expressing Be2C (p = 0.04), while a significant reduction in current density is observed in 6TM-mOR-transfected Be2C cells (p = 0.04). For treated cells 10 μM of morphine were added to growth medium one hour before recordings.

The pharmacological effects of opioids have been associated with an increase in K^+^ conductance, which causes the hyperpolarization of neuronal cells and, ultimately, decreased neuronal excitability [6,37]. Therefore, we investigate the 7TM-mOR- and 6TM-mOR-dependent electrophysiological response in Be2C cells, upon stimulation with morphine. After a one-hour administration of the drug to 7TM-mOR-transfected Be2C cells, we record a rise in an outward current with a reversal potential near -80 mV (Fig 4, panel A), suggesting an enhanced K^+^ conductance, which leads to a decreased cell excitability [6,37]. However, morphine stimulation of 6TM-mOR-transfected Be2C cells leads to a decrease in an outward current with a reversal potential near -80 mV (Fig 4, panel B), suggesting a reduced K^+^ conductance, and, therefore an increased cell excitability [6,37]. Averaged current density data obtained at +50 mV indicates a significant increase in current density in response to morphine in 7TM-mOR-expressing Be2C cells (Fig 4, panel C), whereas a significant reduction in current density at +50 mV is observed in 6TM-mOR-transfected Be2C cells (Fig 4, panel C). Together our results characterize a 6TM-mOR-dependent cellular electrophysiological response, and reveal a reduced morphine-induced K^+^ conductance, which is distinctly different from the morphine-mediated increase of K^+^ conductance observed upon stimulation of 7TM-mOR.

## Conclusions

Our findings point towards a unique morphine-mediated signaling pattern of 6TM-mOR, which is largely different from what has been observed for the major 7TM-mOR isoform. Despite the similarity of binding modes in the two receptors, morphine exhibits the tendency to not activate the same dynamic fluctuations of the i3 loop conformation in 6TM-mOR as observed in 7TM-mOR. As a consequence, and unlike what is observed for the major 7TM-mOR isoform, the stimulation of 6TM-mOR by morphine does not induce a cellular cAMP response. However, a multitude of new and unique morphine-mediated cellular responses are induced, such as the mOR isoform-specific increase of the intracellular Ca^2+^ concentration, and reduced K^+^ conductance, which imply the existence of a mOR isoform specific signaling activity. It is possible that some of the observed signaling differences between the two mOR isoforms may results from their different sub-cellular localization. Indeed, 6TM-mOR is not constitutively expressed on the plasma membrane in mammalian cells, but instead it is mainly localized in intracellular compartments [16,17,26]. Nevertheless, it has been shown that cells overexpressing 6TM-mOR are able to bind labeled naloxone [16]. Additionally, IBNTxA shows high affinity binding to 6TM-mOR only in cells co-transfected with a second GPCR partner (i.e., nociceptin), but not in cells expressing either 6TM-mOR or nociception alone [26]. IBNTxA binding nevertheless is observed in brain tissue extracted from 7TM-mOR knockout mice [26]. Overall, these data indicate that the lack of the first transmembrane helix in 6TM-mOR definitely alters the cellular localization of the receptor. It cannot be excluded that intracellularly localized 6TM-mOR can bind ligands, and initiate cellular signaling like the sigma receptor (Sigma-1) [38] or estrogen receptor GPR30 [39]. However, it is also possible that a yet unidentified chaperone can regulate plasma membrane co-localization, assembly, and 6TM-mOR cellular signaling evoked by opioid ligands. Therefore, the morphine-mediated 6TM-mOR signaling may be related to the previously described physical interactions of this receptor with a second GPCR [18,26]. Thus, it cannot be excluded that the observed cellular responses may be related to the signaling activity of 6TM-mOR in its heterodimeric form with β2AR [18] or nociceptin [26], and to the possible non-canonical opioid receptor signaling pathways that may be consequentely activated through β-arrestin-dependent pathways [11–14]. However, the elucidation of the molecular mechanisms underlying the signaling activity of the heterodimer goes beyond the scope of the presented manuscript and will require further investigation. In conclusion, our data indicate that 6TM-mOR may directly compete with 7TM-mOR for the binding of morphine without generating the same cellular response. Therefore, we foresee 6TM-mOR as a unique player in cellular opioid responses that might constitute a new target for novel pharmacological strategies in pain managements protocols that consider the use of opioid drugs.

## Materials and Methods

### Generation of structural models of 7TM- and 6TM-mOR

We generate the structural models of 7TM- and 6TM-mOR by applying minor changes to the crystallographic coordinates of the 7TM-mOR chimera, as available from the Protein Data Bank [40] (PDB ID: 4DKL) at 2.80 Å resolution [21]. The mouse isoform of mOR (PDB ID: 4DKL) has 93.75% global homology with the human variants, and 100% homology at the level of the binding site and the i3 loop. Therefore, we model 7TM-mOR by removing the coordinates of the co-crystallized morphinan antagonist β-funaltrexamine (β-FNA) and the T4 lysozyme, which replaces the i3 loop between helices 5 and 6 in the opioid receptor. Additionally, in order to obtain the structural model of 6TM-mOR, we remove the first trans-membrane helix (*i*.*e*., from M65 to K98). For both 7TM- and 6TM-mOR models we manually reconstruct the amino acid sequence of the i3 loop (*i*.*e*., from M264 to K269) and model its conformation using discrete molecular dynamics (DMD) [19,20]. In DMD, atomic interactions are approximated by square-well potentials. The simulation engine solves a series of two-body collisions, in which colliding atoms’ velocities change instantaneously according to the conservation laws of energy, momentum, and angular momentum. A united atom representation is used to model protein structure, and the Lazaridis-Karplus implicit solvation model [41] is adopted to account for the solvation energy, while temperature is controlled with the Andersen thermostat [42]. For both 7TM- and 6TM-mOR, we run a short DMD simulation (100,000 steps, *i*.*e*., ~ 5 ns) at the temperature of 0.5 kcal/mol k_B_ (~ 300 K). During the simulation, all atomic coordinates from the crystal structure are harmonically constrained, and only atoms constituting the i3 loop and the connecting peptide bonds are allowed to move freely. We generate a total number of 1,000 i3 loop conformations for 7TM- and 6TM-mOR and, in order to discriminate different conformations, we perform a cluster analysis based on the root mean squared distance (RMSD) of backbone Cα atoms (applied cutoff of 2.5 Å, chosen as a good quality threshold to define high-resolution crystal structures). Finally, we retrieve the centroids of the most populated cluster as the representative conformation of the i3 loop in the 7TM- and 6TM-mOR structural models. We then assess the quality of our generated models using Gaia (http://troll.med.unc.edu/chiron/login.php, [43]), which compares their intrinsic structural properties to high-resolution crystal structures. No critical issues are found in our 7TM- and 6TM-mOR models, which are further used for docking and molecular dynamics studies.

### Docking of morphine in 7TM- and 6TM-mOR

We adopt the obtained structural models of 7TM- and 6TM-mOR for our docking calculations, while structures of ligands are generated using the LigPrep (v. 2.8) module available in Maestro (Schroedinger, LLC New York). Docking calculations are performed using MedusaDock [22,23], our in-house developed software that simultaneously models the flexibility of both ligand and receptor. MedusaDock properly accounts for the induced fit phenomenon upon ligand binding and, thus, it is not sensitive to the starting conformation of amino acids in the GPCR’s binding pocket. Therefore, our docking results are not biased by the starting conformation of the 7TM-mOR binding site in complex with the antagonist β-FNA. During docking, we retrieve representative conformations of morphine in complex with 7TM- and 6TM-mOR by combining the values of MedusaScore [25], a physical force field based scoring function accounting for the protein-ligand interaction energy, with a hierarchical cluster analysis of the top-ranked ligand conformations. In further detail, we run 2,000 independent docking calculations and collect the top-scoring (*i*.*e*., lowest energy) conformations of morphine in complex with 7TM- and 6TM-mOR. We cluster the ensemble of docking solutions according to the RMSD of the ligand’s heavy atoms, and the centroid of the most populated cluster is chosen as the representative conformation of morphine bound to the two receptors. Since MedusaDock does not account for the formation of a covalent bond between ligand and receptor, we are unable to properly evaluate the binding conformation of the co-crystallized molecule β-FNA in a covalent complex with 7TM-mOR. However, we can estimate the performance of our procedure by comparing the relative orientations of the morphinan cores of our docking solutions with respect to the crystallographic pose of β-FNA (panel A S4 Fig). The RMSD computed over the morphinan’s heavy atoms of morphine and β-FNA is 3.8 Å, only 1 Å over the atomic resolution of the crystallographic structure (*i*.*e*., 2.8 Å). Such discrepancy is mainly due to the lack of substituents on the morphinan core of morphine that can deeply penetrate into the 7TM-mOR binding pocket. On the other hand, the bound conformation of the β-FNA is more external because of the steric hindrance exerted by the cyclopropylmethyl substituent and the covalent bond with K233 mediated by the enoate chain. Despite the occurrence of these differences, the relative orientation of the morphinan core is kept in both morphine and β-FNA bound conformations (panel B, S4 Fig). Therefore, the described procedure is able to correctly reproduce the orientation of the morphinan core of opioids in 7TM-mOR binding site as also pointed out by the comparison with previously published docking solutions (panel C S4 Fig and S1 Table, [7]).

### Molecular dynamics simulations of 7TM- and 6TM-mOR

Molecular dynamics (MD) simulations of free and opioid-bound 7TM- and 6TM-mOR are performed using GROMACS [44] and CHARMM36 force field [45], including additional parameters for morphine and lipids. We embed the structures of apo- and morphine-bound opioid isoforms in a lipid bilayer accounting for the presence of water and physiological salt concentration (NaCl). The TIP3P model [46] is employed for explicit water molecules. For the lipid membrane we adopt the 1,2-dipalmitoyl-*sn*-phosphatidylcoline (DPPC) CHARMM36 parameters, which effectively reproduce a series of experimentally determined physicochemical features of lipid bilayers [47]. CHARMM parameters for morphine are derived using the SwissParam website (http://www.swissparam.ch/) [48]. Isothermal-isobaric (NPT) simulations are carried out with a time step of 2 fs and periodic boundary conditions to eliminate the finite size effect (box dimensions 143 x 143 x 162 Å, containing ~ 205,000 atoms). Particle mesh Ewald sum (PME) [49] is employed to model electrostatics, using a 10 Å cutoff distance and 12 Å grid spacing. We adopt the Lennard-Jones switching function over a range of 8 to 12 Å to account for van der Waals interactions, and LINCS algorithm to constrain bond lengths [50]. During the simulation, pressure is set to 1 atm and temperature to 300 K using Parrinello-Rahman barostat [51] and Nosé-Hoover thermostat [52], respectively. To ensure the packing of lipids around the receptors, we impose harmonic constraints on the protein structures and run short simulations (i.e., 0.5 ns each) as described by Serhoijos et al. [7]. For the four systems under investigation, we perform a 20 ns-long equilibration run and, from the last 10 ns of each simulation, we randomly extract three snapshots that are then resubmitted for three further independent 60 ns-long production runs. Finally, the last 40 ns of the MD simulations are taken into account for the analysis of both apo- and morphine-bound 7TM-mOR and 6TM-mOR dynamics.

### cAMP assay

Human HEK293 cells are transiently cotransfected with GloSensor-22F cAMP-sensitive constract and with pIRES-EGFP-7TM-mOR, -6TM-mOR or empty vector (control) expressing constructs, and subjected to cAMP-sensitive luciferase reporter assay (Promega), as described by Serhoijos et al. [7]. Briefly, the day after transfection (24 hr), cells are seeded in 384-well plates. The following day cells pretreated with 100 nM isoproterenol are challenged with morphine at different concentrations and luminescence is recorded on a Victor plate reader (Perkin Elmer). Transfection efficiency is monitored across experiments by EGFP basal level expression, and is comparable in all conditions. We further check the protein expression levels in HEK293 using FLAG-tagged constructs (S5 Fig). However, cAMP and Ca^2+^ experiments (next section) are performed in cells expressing untagged proteins to avoid generation of potentially false results in the case that FLAG interferes with mOR receptor functions.

### Ca^2+^ measurement

Human Be2C cells (ATCC, Manassas, VA, USA) are seeded onto glass coverslips for 24 hours and then transfected with human pIRES-EGFP-7TM-mOR, -6TM-mOR expression constructs or empty vector using Lipofectamine 2000 reagent (Life Technologies Inc., USA) with as described previously [16]. Be2C cells are chosen for this assay because these cells have a robust Ca^2+^ respond and express all cellular components required for its activation. After 24 hours Be2C cells are loaded with 1μM of the cell permeable calcium sensitive dye, Fura-2AM (Life Technologies, USA) for 30 minutes and washed with PBS before imaging. Coverslips containing cells are placed in a chamber with constant infusion of PBS at room temperature as indicated below. Perfusion conditions for Be2C cells are: 1 min PBS; 59 min 10μM morphine (NIH/NIDA, USA). Fluorescence is detected by a Nikon Eclipse TI microscope at 340 and 380 nm wavelengths and analyzed with the TI Element Software (Nikon, Japan). Cells are considered as responsive to a drug infusion if the 340/380 ratio is ≥ 0.2 from baseline. Mean values are compared using one-ANOVA and Fisher’s LSD. Expression levels of 7TM-, and 6TM-mOR in Be2C cells are comparable with what previously characterized by Gris et al. using RT-PCR [16].

### Electrophysiology measurements

One day prior to transfection, Be2C cells are plated at ~50% confluency onto 35 mm plastic dishes. Cells are transiently transfected with a 7TM-mOR-containing eGFP plasmid, 6TM-mOR-containing eGFP plasmid, or an empty eGFP control plasmid using FuGene 6 (Promega). Transfections are carried out according to manufacturer’s instructions using 2 μg of DNA. Cells co-transfected with 6TM-mOR, and the β2AR receptor-tdTomato fusion gene, receive 1 μg of each plasmid. One day following transfection, cells are plated onto 35 mm glass bottom dishes. Whole-cell patch clamp recordings are made 48 hours after transfection. External solution contains: 140 mM NaCl, 3 mM KCl, 1 mM MgCl_2_, 10 mM HEPES, 2 mM CaCl_2_, and 10 mM glucose (pH 7.4, 294 mOsm). The internal pipette solution consists of 123 mM K-gluconate, 10mM KCl, 1 mM MgCl_2_, 10 mM HEPES, 1 mM EGTA, 0.1 mM CaCl_2_, 1 mM K_2_ATP, 0.2 mM Na_4_ATP, and 4 mM glucose (pH 7.2, 302 mOsm). For treated cells, morphine (10 μM) is added to growth medium 1 hour before recordings. Current-voltage (I-V) plots are determined by recording the current response to voltage steps ranging from -90 to +50 mV in 10 mV increments from a holding potential of -70 mV. All recordings are performed with an Axon MultiClamp 700B amplifier (Molecular Devices) using non-coated glass pipettes (2.5–5 MΩ). Clampex 10.3 (Molecular Devices) is used for data acquisition. Data analysis and figure preparation was performed using Clampfit 10.3 (Molecular Devices) and Microsoft Excel.

## Supporting Information

S1 Fig Inter-atomic distances between morphine and 7TM-mOR. Average (in black) and standard deviation (cyan) of distances between atoms of morphine (in red at the top) and amino acids in 7TM-mOR binding site are reported. Specifically, from top to bottom in the left column: morphine-C14-A117-Cβ, morphine-N11-D147-Cγ, morphine-N150-Cγ, morphine-M151-Cβ, and morphine-C2-W293-Cβ. From top to bottom in the right column: morphine-C3-I296-Cβ, morphine-O21-H297-Nδ, morphine-C16-I322-Cβ, morphine-C15-G235-Cα, and morphine-C8-Y236-Cβ. Analyses are performed on the last 40 ns of simulations of three independent simulations.
(PDF)

S2 Fig Inter-atomic distances between morphine and 6TM-mOR. Average (in black) and standard deviation (cyan) of distances between atoms of morphine (in red at the top) and amino acids in 6TM-mOR binding site are reported. Specifically, from top to bottom in the left column: morphine-C14-A117-Cβ, morphine-N11-D147-Cγ, morphine-N150-Cγ, morphine-M151-Cβ, and morphine-C2-W293-Cβ. From top to bottom in the right column: morphine-C3-I296-Cβ, morphine-O21-H297-Nδ, morphine-C16-I322-Cβ, morphine-C15-G235-Cα, and morphine-C8-Y236-Cβ. Analyses are performed on the last 40 ns of simulations of three independent simulations.
(PDF)

S3 Fig Similarity of morphine bound conformations in 7TM- and 6TM-mOR. **A)** Superimposition of morphine binding mode in 7TM- (green) and 6TM-mOR (blue) as obtained with MedusaDock (t = 0 ns). The energy of binding, estimated using MedusaScore, is equal to -40.3 kcal/mol and -39.5 kcal/mol for 7TM-mOR and 6TM-mOR, respectively. The RMSD of superimposed conformations is ~ 0.3 Å. **B)** Superimposition of the lowest energy bound conformations of morphine in 7TM- (green) and 6TM-mOR (blue) obtained from three independent MD simulations. No clustering analysis has been performed for the selection of bound conformations because of the persistence of morphine coordinates within the crystallographic resolution of the receptor along the entire MD simulation (*i*.*e*., 2.8 Å, Fig 1A). The energy of binding of the final conformations (t = 60 ns), estimated using MedusaScore, is equal to -42.9 kcal/mol and -46.5 kcal/mol for 7TM-mOR and 6TM-mOR, respectively. The RMSD of superimposed conformations is ~ 1.3 Å. Residue labels are reported only in the left figure.
(PDF)

S4 Fig Morphinan core orientation of morphine and β-FNA in complex with 7TM-mOR. **A)** Chemical structures of morphine (left) and β-FNA (right), in which the morphinan core is highlighted in red. **B)** Superimposition of MedusaDock docking solution of morphine (green) to the crystallographic conformation of β-FNA covalently bound to K233 in 7TM-mOR binding site. **C)** Superimposition of previously published docking solution of morphine [7] (orange) to the crystallographic conformation of β-FNA covalently bound to K233 in 7TM-mOR binding site. In (B) and (C) mOR electron density map as available from the Electron Density Server (ref. [53] in the main text) is reported as white mesh.
(PDF)

S5 Fig Western blot analysis of HEK293 cell total lysates using antibodies against FLAG tag. Cells were transiently transfected with FLAG-tagged 7TM-, 6TM-mOR, or empty vector. 48h after cells were lysed with RIPA lysis buffer (ThermoFisher Scientific); protein concentrations were determined with the BCA protein assay kit (ThermoFisher Scientific) and 20 μg lysates were loaded per lane for a SDS-PAGE gel separation. WB: probing antibody.
(PDF)

S1 Table Root mean square distances among docking solutions. (DOCX)
