# Supplementary figures and images for: Differential Regulation of 6- and 7-Transmembrane Helix Variants of μ-Opioid Receptor in Response to Morphine Stimulation
Source: PLoS One. 2015 Nov 10;10(11):e0142826. doi: 10.1371/journal.pone.0142826 (PMC4640872; doi:10.1371/journal.pone.0142826)

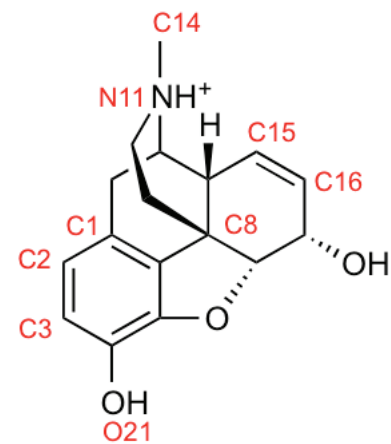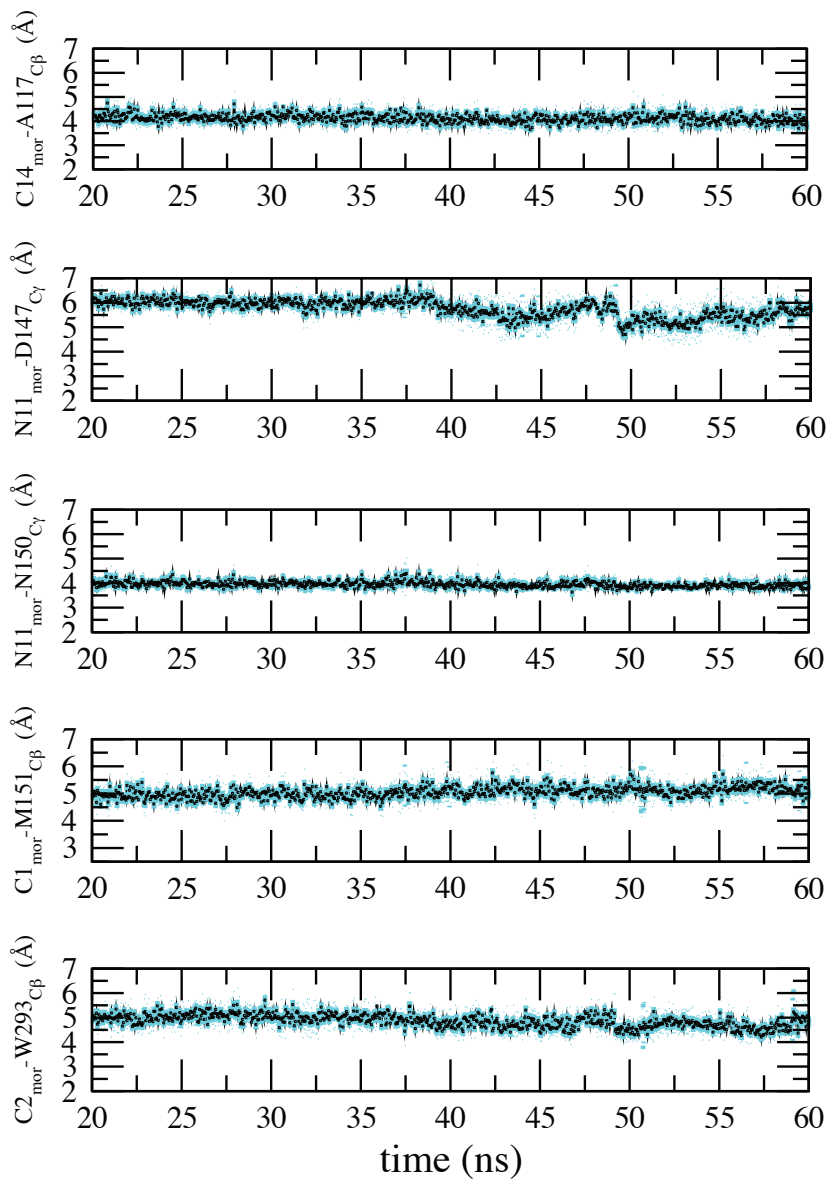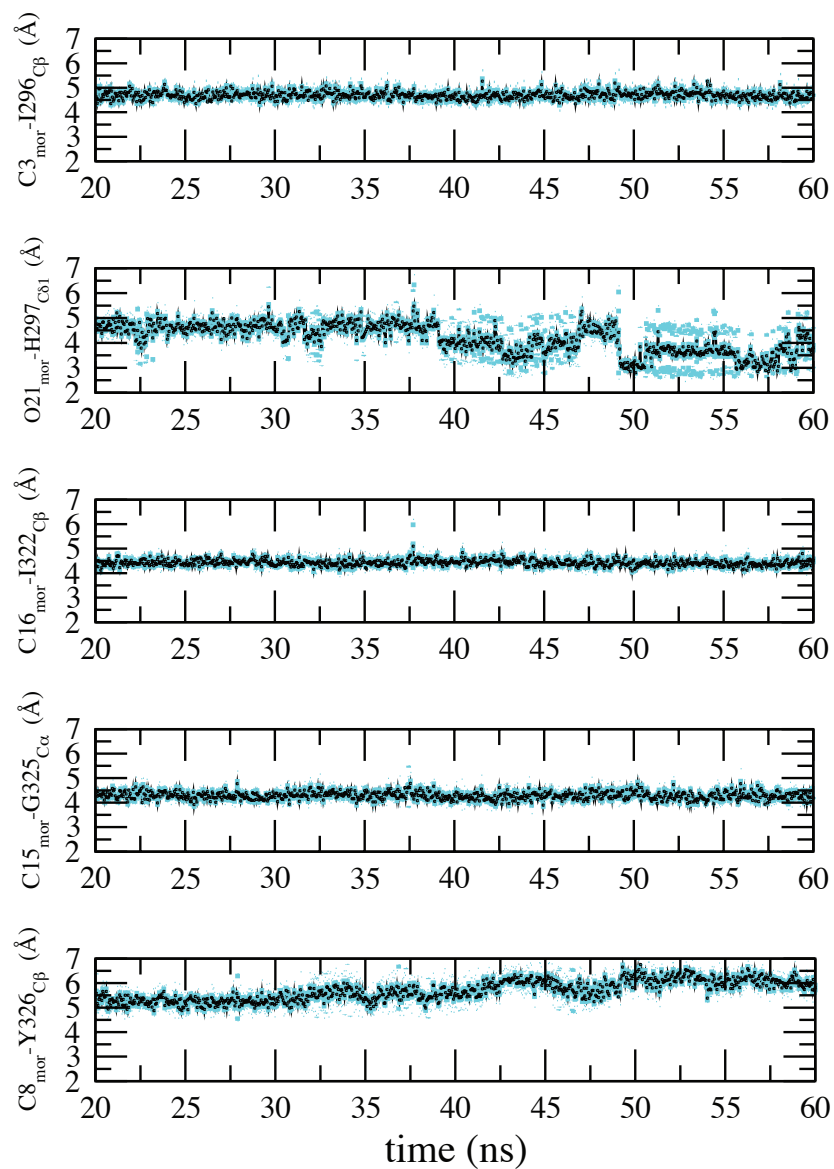

Supplement: S1 Fig — Average (in black) and standard deviation (cyan) of distances between atoms of morphine (in red at the top) and amino acids in 7TM-mOR binding site are reported. Specifically, from top to bottom in the left column: morphine-C14-A117-Cβ, morphine-N11-D147-Cγ, morphine-N150-Cγ, morphine-M151-Cβ, and morphine-C2-W293-Cβ. From top to bottom in the right column: morphine-C3-I296-Cβ, morphine-O21-H297-Nδ, morphine-C16-I322-Cβ, morphine-C15-G235-Cα, and morphine-C8-Y236-Cβ. Analyses are performed on the last 40 ns of simulations of three independent simulations. (PDF) [file pone.0142826.s001.pdf]

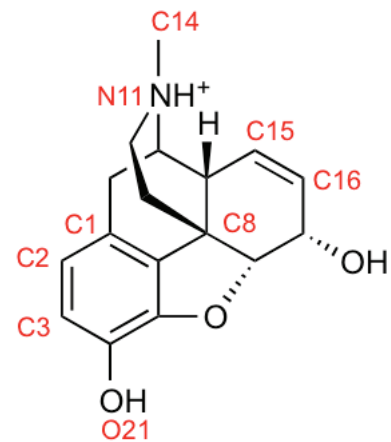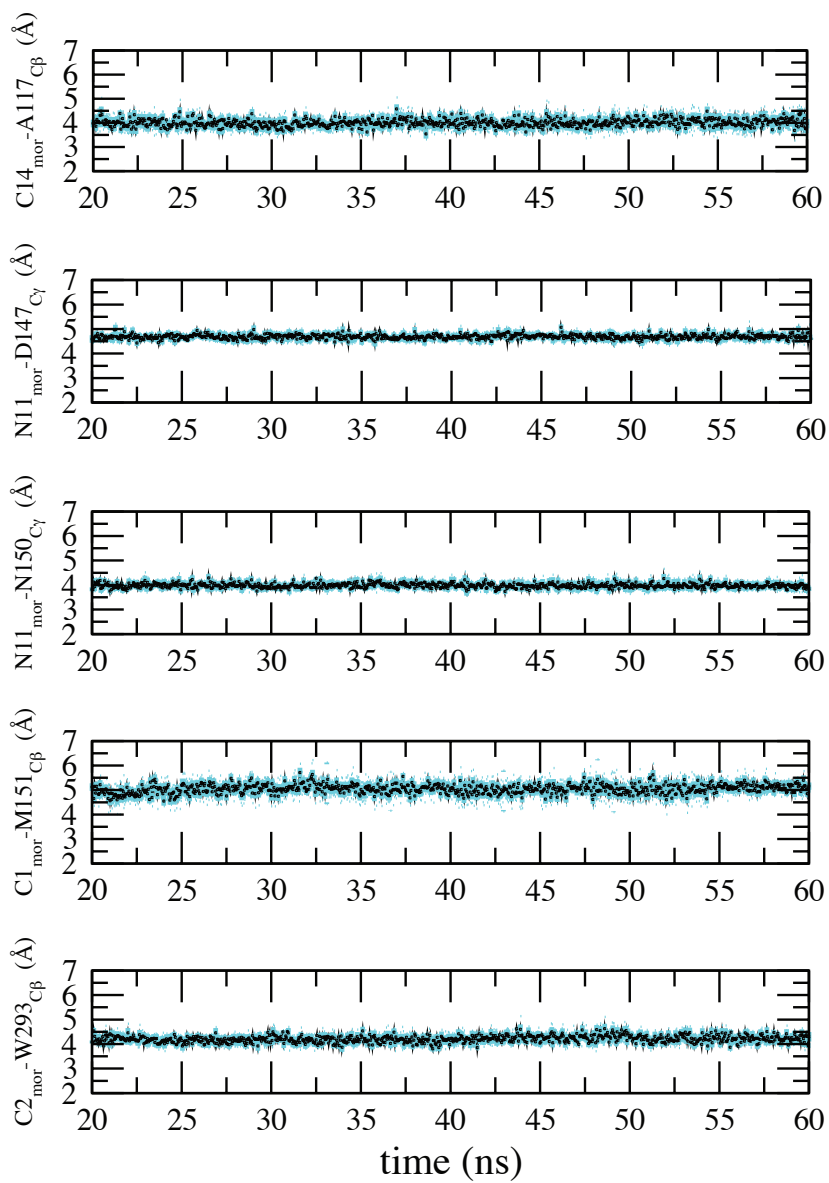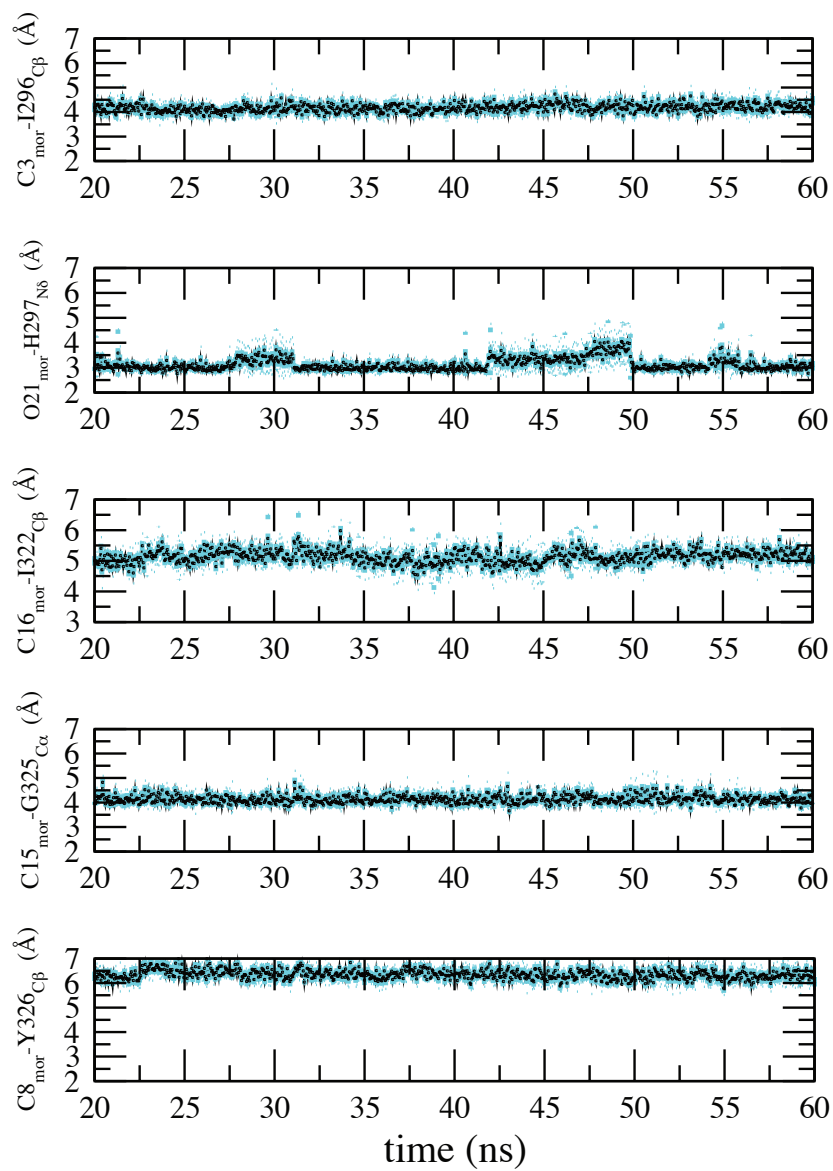

Supplement: S2 Fig — Average (in black) and standard deviation (cyan) of distances between atoms of morphine (in red at the top) and amino acids in 6TM-mOR binding site are reported. Specifically, from top to bottom in the left column: morphine-C14-A117-Cβ, morphine-N11-D147-Cγ, morphine-N150-Cγ, morphine-M151-Cβ, and morphine-C2-W293-Cβ. From top to bottom in the right column: morphine-C3-I296-Cβ, morphine-O21-H297-Nδ, morphine-C16-I322-Cβ, morphine-C15-G235-Cα, and morphine-C8-Y236-Cβ. Analyses are performed on the last 40 ns of simulations of three independent simulations. (PDF) [file pone.0142826.s002.pdf]

A

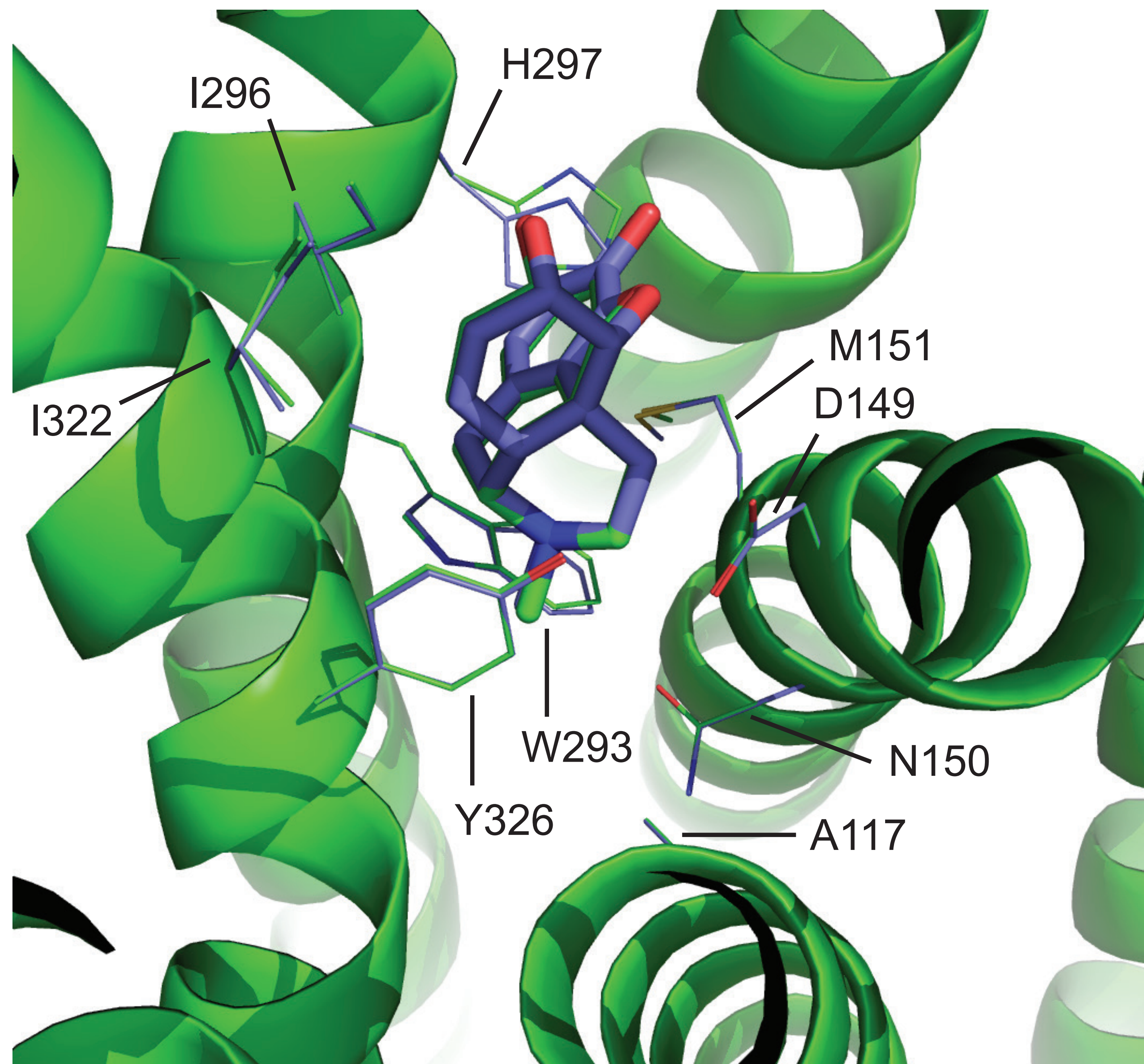

B

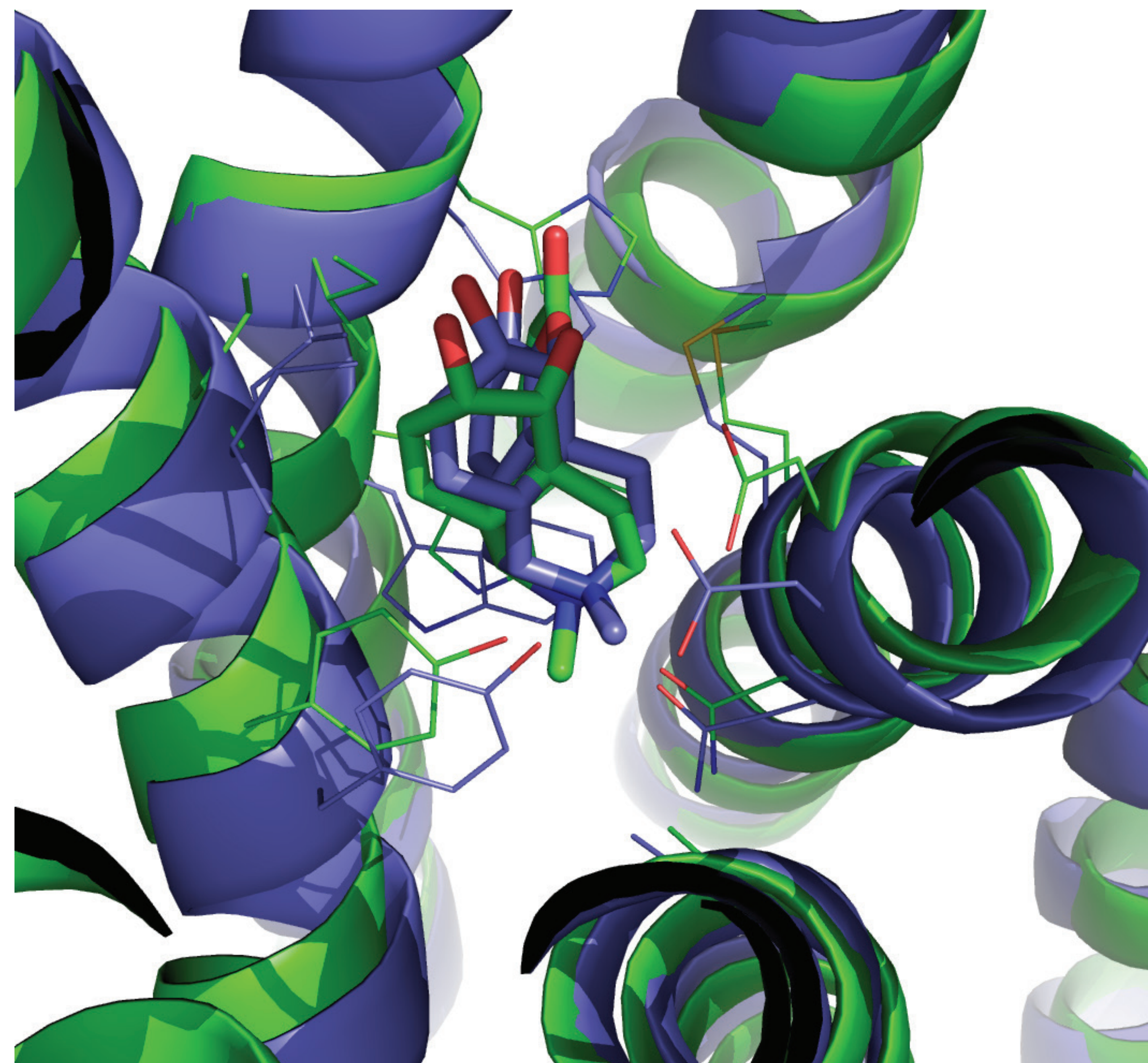

Supplement: S3 Fig — A) Superimposition of morphine binding mode in 7TM- (green) and 6TM-mOR (blue) as obtained with MedusaDock (t = 0 ns). The energy of binding, estimated using MedusaScore, is equal to -40.3 kcal/mol and -39.5 kcal/mol for 7TM-mOR and 6TM-mOR, respectively. The RMSD of superimposed conformations is ~ 0.3 Å. B) Superimposition of the lowest energy bound conformations of morphine in 7TM- (green) and 6TM-mOR (blue) obtained from three independent MD simulations. No clustering analysis has been performed for the selection of bound conformations because of the persistence of morphine coordinates within the crystallographic resolution of the receptor along the entire MD simulation (i.e., 2.8 Å, Fig 1A). The energy of binding of the final conformations (t = 60 ns), estimated using MedusaScore, is equal to -42.9 kcal/mol and -46.5 kcal/mol for 7TM-mOR and 6TM-mOR, respectively. The RMSD of superimposed conformations is ~ 1.3 Å. Residue labels are reported only in the left figure. (PDF) [file pone.0142826.s003.pdf]

A

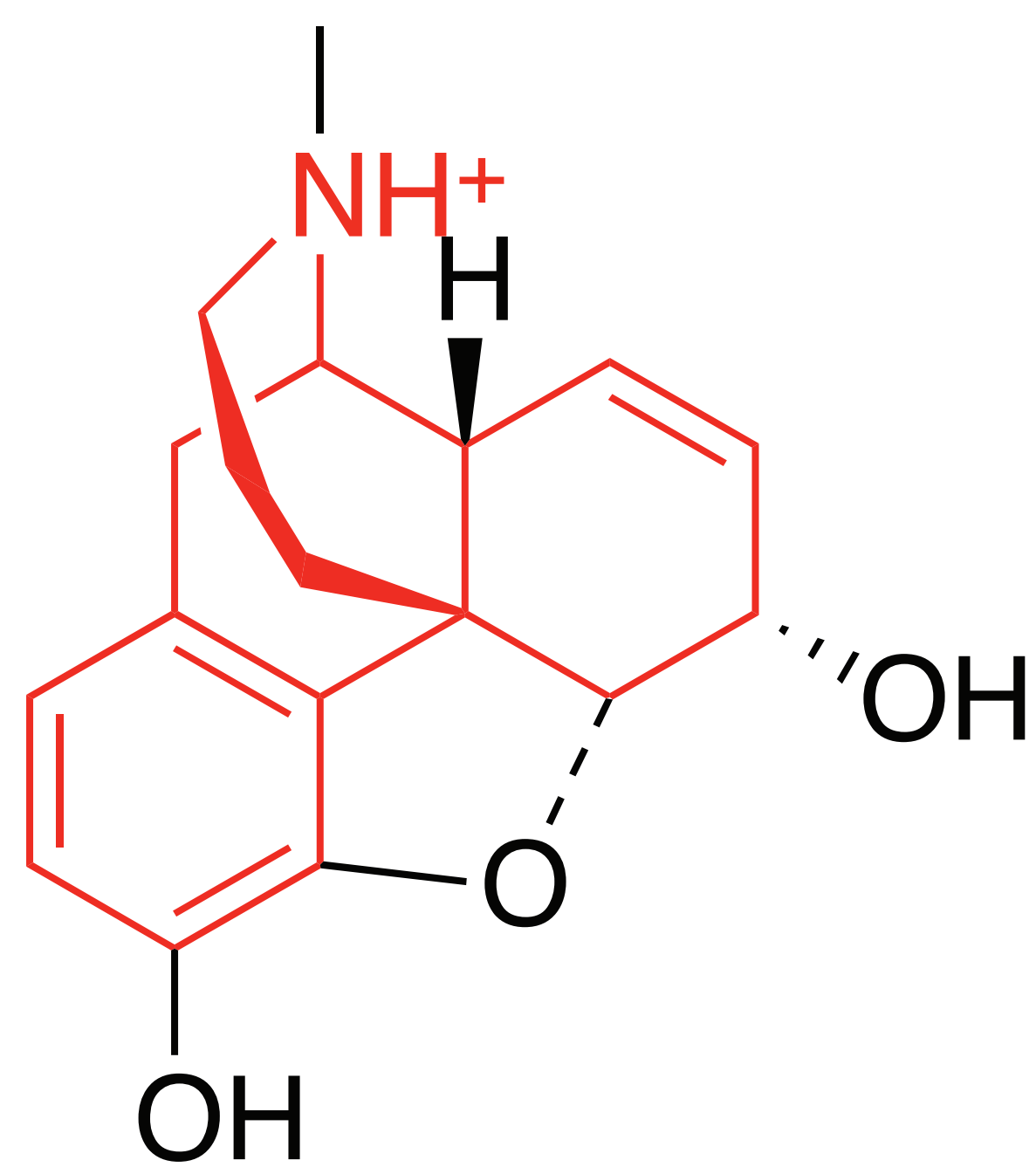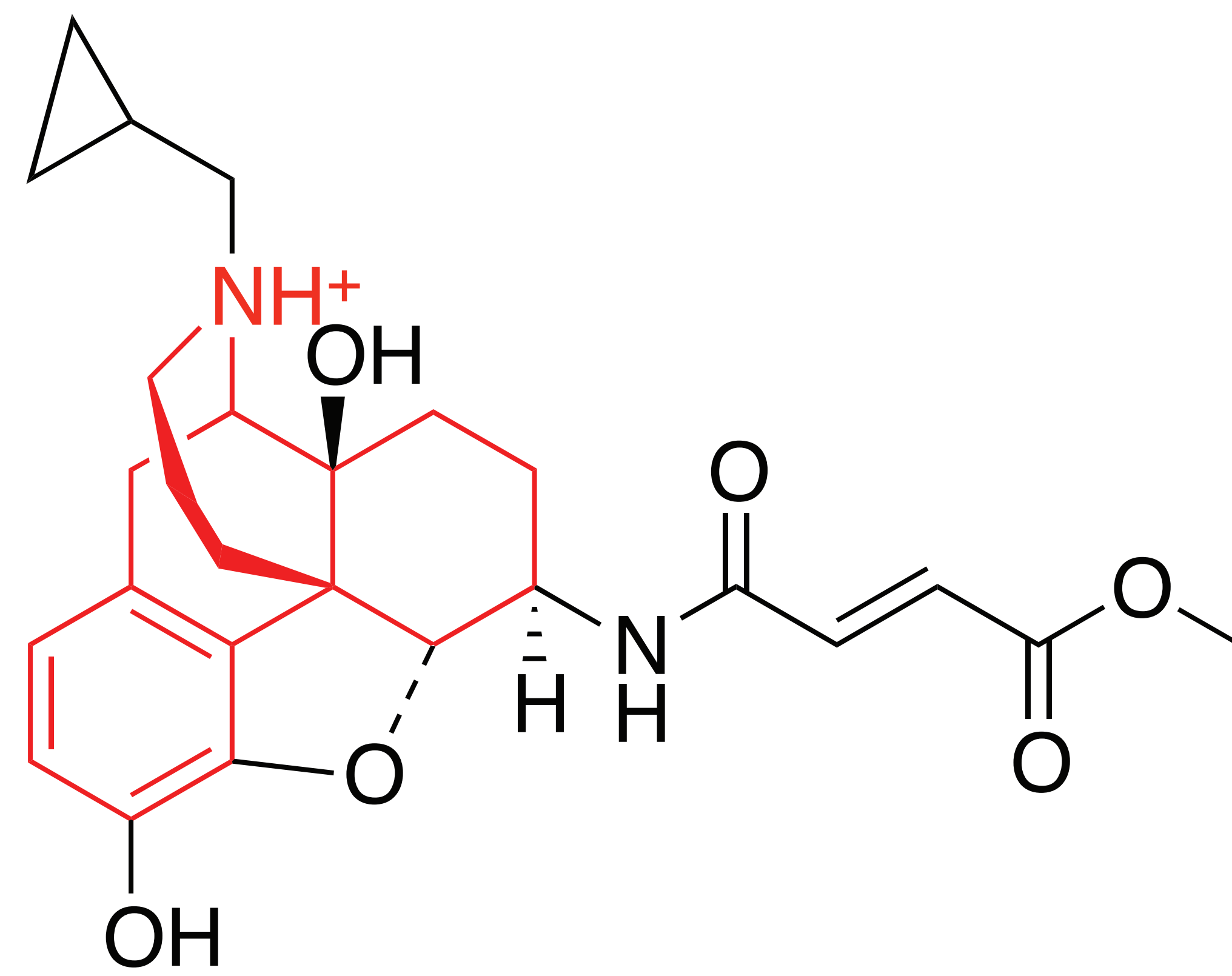

B

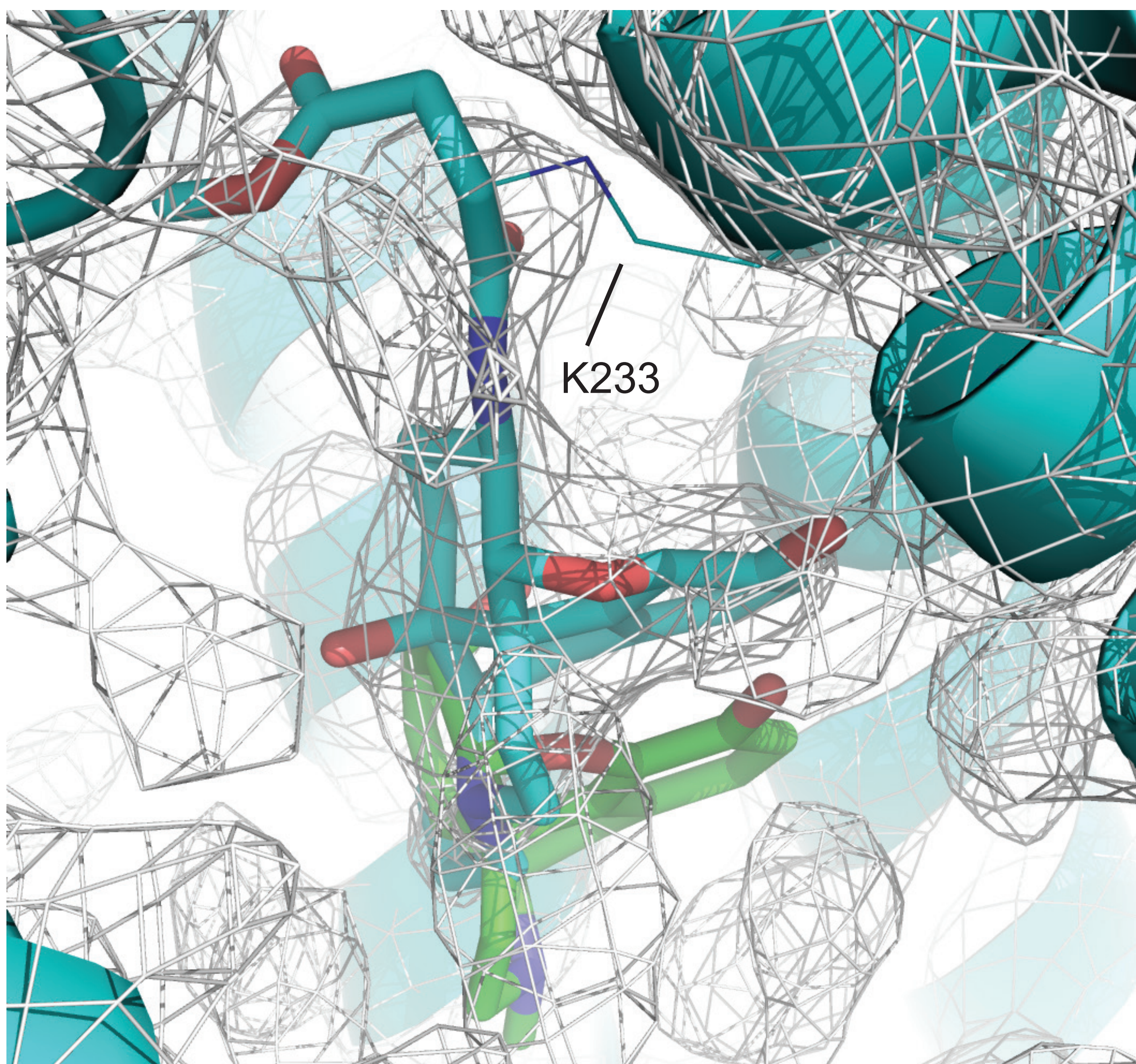

C

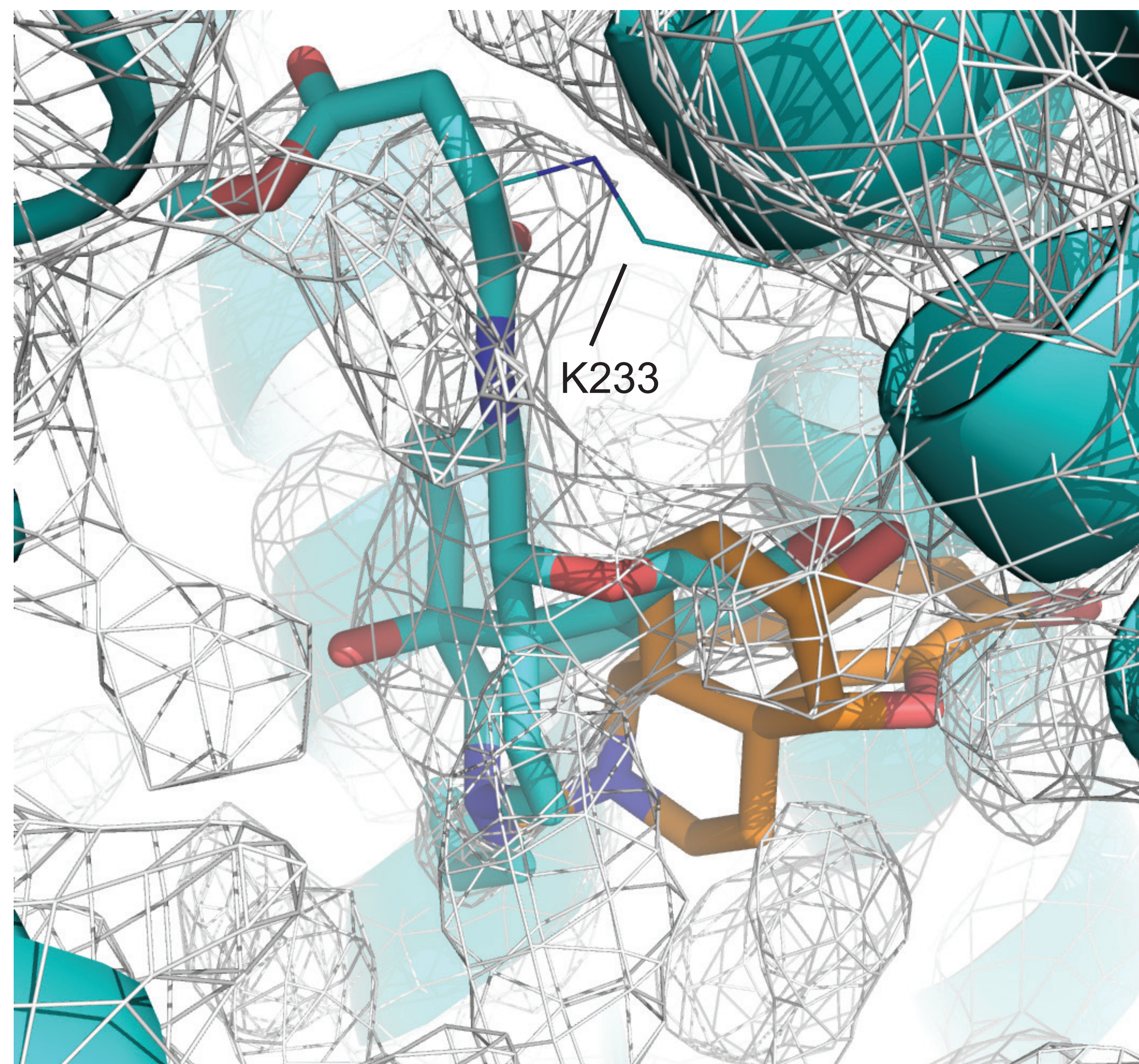

Supplement: S4 Fig — A) Chemical structures of morphine (left) and β-FNA (right), in which the morphinan core is highlighted in red. B) Superimposition of MedusaDock docking solution of morphine (green) to the crystallographic conformation of β-FNA covalently bound to K233 in 7TM-mOR binding site. C) Superimposition of previously published docking solution of morphine [7] (orange) to the crystallographic conformation of β-FNA covalently bound to K233 in 7TM-mOR binding site. In (B) and (C) mOR electron density map as available from the Electron Density Server (ref. [53] in the main text) is reported as white mesh. (PDF) [file pone.0142826.s004.pdf]

WB: Anti-FLAG

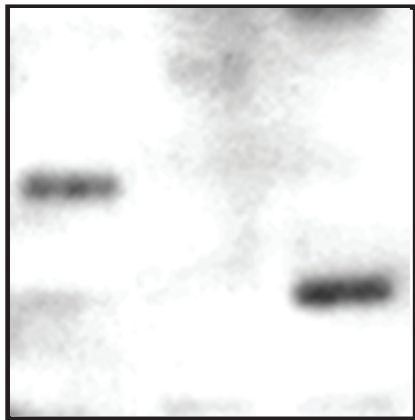

← ~50 kDa

← ~35 kDa

7TM-mOR

Empty Vector

6TM-mOR

Supplement: S5 Fig — Cells were transiently transfected with FLAG-tagged 7TM-, 6TM-mOR, or empty vector. 48h after cells were lysed with RIPA lysis buffer (ThermoFisher Scientific); protein concentrations were determined with the BCA protein assay kit (ThermoFisher Scientific) and 20 μg lysates were loaded per lane for a SDS-PAGE gel separation. WB: probing antibody. (PDF) [file pone.0142826.s005.pdf]
