# Supplementary material for: Differential Regulation of 6- and 7-Transmembrane Helix Variants of μ-Opioid Receptor in Response to Morphine Stimulation
Source: PLoS One. 2015 Nov 10;10(11):e0142826. doi: 10.1371/journal.pone.0142826 (PMC4640872; doi:10.1371/journal.pone.0142826)
Supplement: S1 Table — (DOCX) [file pone.0142826.s006.docx]

**Table S1.** **Root mean square distances among docking solutions.**

| **Morphinan core RMSD (Å)** | **MedusaDock** | **Previous Docking^*^** | **β-FNA** |
| --- | --- | --- | --- |
| **MedusaDock** | 0 | 5.7 | 3.8 |
| **Previous Docking** | 5.7 | 0 | 4.7 |
| **β-FNA** | 3.8 | 4.7 | 0 |

Values are computed over the morphinan core’s heavy atoms of the MedusaDock solution, the previously published docking solutions for morphine,^*^ and the crystallographic conformation of β-FNA covalently bound to 7TM-mOR. ^*^Ref. [7] in the main text.
